# Transcription of the extended *hyp*-operon in *Nostoc *sp. strain PCC 7120

**DOI:** 10.1186/1471-2180-8-69

**Published:** 2008-04-28

**Authors:** Åsa Agervald, Karin Stensjö, Marie Holmqvist, Peter Lindblad

**Affiliations:** 1 Department of Photochemistry and Molecular Science, The Ångström Laboratories, Uppsala University, Box 523, SE-751 20 Uppsala, Sweden

## Abstract

**Background:**

The maturation of hydrogenases into active enzymes is a complex process and e.g. a correctly assembled active site requires the involvement of at least seven proteins, encoded by *hypABCDEF *and a hydrogenase specific protease, encoded either by *hupW *or *hoxW*. The N_2_-fixing cyanobacterium *Nostoc *sp. strain PCC 7120 may contain both an uptake and a bidirectional hydrogenase. The present study addresses the presence and expression of *hyp*-genes in *Nostoc *sp. strain PCC 7120.

**Results:**

RT-PCRs demonstrated that the six *hyp*-genes together with one ORF may be transcribed as a single operon. Transcriptional start points (TSPs) were identified 280 bp upstream from *hypF *and 445 bp upstream of *hypC*, respectively, demonstrating the existence of several transcripts. In addition, five upstream ORFs located in between *hupSL*, encoding the small and large subunits of the uptake hydrogenase, and the *hyp*-operon, and two downstream ORFs from the *hyp*-genes were shown to be part of the same transcript unit. A third TSP was identified 45 bp upstream of asr0689, the first of five ORFs in this operon. The ORFs are annotated as encoding unknown proteins, with the exception of alr0692 which is identified as a NifU-like protein. Orthologues of the four ORFs asr0689-alr0692, with a highly conserved genomic arrangement positioned between *hupSL*, and the *hyp *genes are found in several other N_2_-fixing cyanobacteria, but are absent in non N_2_-fixing cyanobacteria with only the bidirectional hydrogenase. Short conserved sequences were found in six intergenic regions of the extended *hyp*-operon, appearing between 11 and 79 times in the genome.

**Conclusion:**

This study demonstrated that five ORFs upstream of the *hyp*-gene cluster are co-transcribed with the *hyp*-genes, and identified three TSPs in the extended *hyp*-gene cluster in *Nostoc *sp. strain PCC 7120. This may indicate a function related to the assembly of a functional uptake hydrogenase, hypothetically in the assembly of the small subunit of the enzyme.

## Background

The global energy demand will increase drastically in the near future due the population and economic growth; calculations indicate at least a 2-fold increase over 50 years [1]. To be able to meet the global energy demand new and sustainable non-coal based or carbon-neutral energy resources have to be developed. Molecular hydrogen, H_2_, is one of the upcoming promising alternative energy carriers [2]. Cyanobacteria are together with green algae possible candidates for future clean and sustainable energy production of hydrogen, since they are the only organisms capable of the unique combination of having oxygenic photosynthesis and hydrogenases, allowing them to use and convert solar energy and water into hydrogen (H_2_) [3-9].

The enzymes directly involved in hydrogen metabolism in cyanobacteria are hydrogenases and nitrogenases. Depending on the cyanobacterial strain, a single cell can harbour either an uptake hydrogenase or a bidirectional enzyme or both. N_2_-fixing strains contain at least an uptake hydrogenase which recycles the energy rich hydrogen molecule is produced as a by-product of the nitrogenase under N_2_-fixation. Hydrogenases, as well as nitrogenases, are very sensitive to oxygen which inactivates the activity of the proteins. To protect these enzymes they are physically located in the special cell type called heterocyst [5,6,10], or function under anaerobic conditions only [7,11].

The cyanobacterial uptake hydrogenase consists of at least two functional subunits, encoded by the structural genes *hupL *and *hupS*. HupL harbours the active site and HupS harboururs iron-sulphur (FeS) slusters which transfer electrons from the active site [12].

All cyanobacterial hydrogenases are classified as NiFe-hydrogenases, being either uptake hydrogenases (HupSL) or bidirectional hydrogenases (HoxEFUYH), [5,6,8,9,12]. The active site has a complex structure with one Ni and one Fe atom, to which biochemically unusual ligands of CN and CO are bound. In order to develop a fully active and mature hydrogenase at least seven specific proteins are required. Of the seven, the six Hyp proteins encoded by *hypABCDEF *(*hyp *for *hy*drogenase *p*leiotropic) are responsible for the insertion of the metal atoms into the acitve site of the hydrogenases, as well as the attachement of the ligands to the Fe atom [5,6,12,13]. The function of the *hyp*-genes have been mainly studied in *E. coli*. The high homology to the cyanobacterial *hyp*-genes indicates that the role is the same in cyanobacteria. Indeed, analyses of deletion and insertion mutants of *hyp *genes in *Synechocystis *sp. PCC 6803 showed no hydrogenase activity [14]. The *hyp*-genes are conserved and can either be found together, e.g. in *Nostoc *sp. strain PCC 7120 and *Anabaena variabilis *ATCC 29413 or spread out in the genome as in *Synechosystis *sp. strain PCC 6803. There is only one set of *hyp*-genes independent of the number of hydrogenases in the cells [14]. This indicates a co-regulation of the *hyp*-genes on the assembly of both types of hydrogenases [5,15]. How this is achieved is not known, but the *hyp*-genes should be regulated differently depending on e.g. strains, environment and type of hydrogenase. The seventh factor is encoded either by *hupW *or *hoxW*, hydrogenase specific proteases which are needed to cleave off part of the C-terminal of the large subunit [13]. This is only done after the insertion of Ni in the active site and may function as a checkpoint for the maturation process [16,17]. The cleavage enables a conformal change of the large subunit, which is necessary for the binding of the small subunit, HupS.

The small subunit of hydrogenases harbours (FeS) clusters which are the main components in electron transport to and from the active site and they define the electron transport pathways in both membrane-bound and soluble redox-enzymes [12]. How the assembly and maturation process is achieved is not well known, but three different types of (FeS) cluster assembly have been presented with two requirements in common: the need for a (FeS) scaffolding protein, and a cysteine desulphurase which is required to yield elemental sulphur or hydrogen sulphide [12,18].

*Nostoc *sp. strain PCC 7120 is a N_2_-fixing filamentous and heterocyst-forming cyanobacterium. The 7.21 Mb genome contains a single nitrogenase, and both an uptake and a bidirectional hydrogenases are present [19,20].

To improve the yield of H_2 _produced by cyanobacteria and e.g. to establish the foundation for the introduction of foreign hydrogenases into cyanobacteria it is essential to understand the regulation of the genes directly involved in the maturation of cyanobacterial hydrogenase. In the present paper we describe the transcriptional regulation of the *hyp*-genes and neighbour open reading frames in *Nostoc *sp. strain PCC 7120. We also discuss the putative function of the upstream genes and the role for the conserved sequences present in some of the intergenic regions of the extended *hyp*-operon.

## Results

### Transcription of the extended hyp-operon

**Figure 1:**
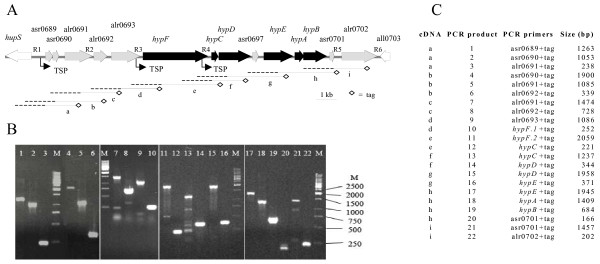
**(A) Physical map of the extended *hyp*-operon, covering a region of almost 14 kb, in *Nostoc *sp. strain PCC 7120.** Apart from the *hyp*-genes (depicted in black) the region also includes five genes upstream of *hypF *(depicted in grey), the two genes downstream of *hypB *(depicted in grey), and asr0697 (between hypD and hypE, depicted in grey). The overlapping cDNA of the region are marked a-i. Identified transcriptional start points, TSP, of the extended *hyp*-operon located upstream of *asr0689*, *hypF *and *hypC *are marked with arrows. Number R1–R6 represents the intergenic regions where conserved sequences were found in the extended *hyp*-operon. (B) Agarose gel showing the overlapping amplified PCR products, 1–22, of the RT-PCR experiments, a-i. (C) Primer pairs (1–22) used in the mapping of the extended *hyp*-operon consisting of overlapping cDNA (a-i). The size indicates the expected length of the PCR-product of the forward primer together with the reverse tag-primer.

To determine if the *hyp*-genes are transcribed as a single operon and to identify putative 5'RACE transcriptional start points (TSPs), reverse transcriptase PCR (RT-PCR), Northern blot, and experiments were performed using total RNA isolated from N_2_-fixing cultures. The six *hyp*-genes, *hypFCDEAB*, the ORF asr0697 located between *hypD *and *hypE*, the two downstream ORFs and five upstream ORFs are all shown to be part of the same operon (Fig. 1A–C). To eliminate any false results from contaminating genomic DNA a specially designed tag was used in the RT-PCR reactions [21] (see Table 1). To cover the complete 14 kb sequence overlapping cDNAs of 2 kb were synthesized (Fig. 1A–C). The four upstream ORFs asr0689, asr0690, alr0691, and alr0693, encode unknown or hypothetical proteins, and alr0692 is annotated as a gene encoding a protein similar to NifU. All proteins have conserved domains; asr0689 and asr0690 encodes possible ABC-transporters with membrane spanning regions, alr0691 encodes a protein with TPR (Tetratrico Peptide Repeats) and prenyltransferase like domains, alr0692 encodes a protein containing NifU and thioredoxin domains, and the protein product of alr0693 harbours NHL (NCL-1, HT2A and Lin-41) and TPR repeats (Table 2).

**Table 1:** Oligonucleotides used in the RT-PCR, PCR, Northern blot, and 5'RACE experiments

Gene	RT-primer sequence 5'-3'	Forward primer sequence 5'-3'	Reverse primer 5'-3
RT-PCR AND PCR			
asr0689	-	tggttagttgtagccacgctta	tag
asr0690	-	cggcgaggttatttttgaag	tag
alr0691	tag + ctggggtggtcaatcaagtt	ttggcgaggataaccgatag	tag
alr0692	tag + cttgggataacgtcaaagtagaa	aaaagggcgattgaggattt	tag
alr0693	tag + tacccttagcattctg	tgggacaaaggaatttttcg	tag
*hypF.1*	tag + ccttgatatggtgtcctgat	cgactgaggaaattcgagtg	tag
*hypF.2*	tag + ttcatcaccacatacc	aagggaattggtggttttca	tag
*hypC*	tag + gcggcgatttcttgtaataat	tcaccgacattaaccacaaa	tag
*hypD*	-	ccaattgttgtttccggttt	tag
*hypE*	tag + tcgtccttttggatgagattt	attttaattgcccgtggtga	tag
*hypA*	-	tgaatatgctcaaggctcaaaa	tag
*hypB*	tag + tggaagcatccaaatgacaa	gggaacaggttgtcatttgg	tag
asr0701	tag + gctgggaaagcttgatatat	acgctattacatcaaattct	tag
alr0702	tag + taaaccgctattggggtcag	tggtgaagtgattgggatga	tag
			
NORTHERN BLOT			
asr0689		tggttagttgtagccacgctta	cccagacccaaaacaatagc
alr0693		tgggacaaaggaatttttcg	aaaacttttctgccccaaca
*hypF*		aagggaattggtggttttca	tcaaatgatgcaaaggcgta
*hypA-hypB*		tgaatatgctcaaggctcaaaa	tgtgggggtatttgattggt
			
IDENTIFICATION OF TRANSCRIPTION START POINTS, 5'RACE			
asr0689	taagcgtggctacaactaacca		
alr0693	tcaacaaccgacgattacca		
*hypF*	cagcatccacatcatccaac		
*hypC*	agcccaaagcgaagaataca		

tag = CACACCACAACCACACGAC

**Table 2:** Annotation, and functional domains of the deduced proteins, of the open reading frames (ORFs) found in the extended *hyp*-operon of *Nostoc *sp. strain PCC 7120 and the closest homologues in *Anabaena variabilis *ATCC 29413 (Fig. 1A) [45]

ORF	Annotation of gene product in *Nostoc* sp. strain PCC 7120	Functional domain/-s of deduced gene product	Closest homologues ATCC 29413 (% aa sequence identity of deduced gene product)	References [46, 47]
asr0689	unknown protein	2 transmembrane regions	ava_ 4597 (94.3)	UniProtKB/TrEMBL-Q8YZ01, IPR003439
asr0690	unknown protein	2 transmembrane regions	ava_4598 (98.6)	UniProtKB/TrEMBL:Q8YZ00, IPR003439
alr0691	hypothetical protein	TPR, Protein prenyltransferase	ava_4599 (97.5)	UniProtKB/TrEMBL-Q9WWQ1, IPR001440/PF00515, IPR008940
alr0692	similar to NifU protein	NifU (C-terminal), Thioredoxin-like domains	ava_4600 (95.6)	UniProtKB/TrEMBL-Q8YYZ9, PF01106, COG0694
alr0693	unknown protein	NHL and TPR repeats	ava_4601 (96.9)	UniProtKB/TrEMBL-Q8YYZ8, Q9WWQ1, IPR001258/PF01436
*hypF*	hydrogenase maturation protein	Hydrogenase expression/formation protein (HUPF/HYPC)	ava_4602 (91.5)	
*hypC*	hydrogenase expression/formation protein	Hydrogenase expression/formation protein (HUPF/HYPC)	ava_4603 (96.3)	
*hypD*	hydrogenase expression/formation protein	Hydrogenase formation HypD protein	ava_4604 (95.6)	
asr0697	probable 4-oxalocrotonate tautomerase	4-oxalocrotonate tautomerase	ava_4605 (98.6)	
*hypE*	hydrogenase expression/formation protein	Hydrogenase expression/formation protein HypE	ava_4606 (98.1)	
*hypA*	hydrogenase expression/formation protein	Hydrogenase expression/synthesis protein, HypA	ava_4607 (89.4)	
*hypB*	hydrogenase expression/formation protein	[NiFe]-hydrogenase/urease maturation factor, Ni(2^+^)-binding GTPase	ava_4608 (88.9)	
asr0701	unknown protein	1 transmembrane region	ava_4178 (44.0) & alr1571 (46.0) ^a^	
alr0702	serine proteinase	Serine proteinase	ava_4610 (96.9)	

^a ^The orthologue, alr1571, present in *Nostoc *sp. strain PCC 7120 has an amino acid sequence identity of 46% to asr0701 and an aa sequence identity of 99% compared with ava_4178.

The open reading frame asr0697, located between *hypD *and *hypE*, is annotated as encoding a probable 4-oxalocrotonate tautomerase and has an orthologue in *Anabaena variabilis *ATCC 29413 with an amino acid sequence identity of 98.6%. The two ORFs asr0701 and alr0702, positioned downstream of the *hyp*-cluster, are encoding proteins with unknown function and as being a serine proteinase, respectively. asr0701 has homologues in both *Nostoc *sp. strain PCC 7120 and *Anabaena variabilis *ATCC 29413 where the encoded proteins share an amino acid sequence identity of 46% with the gene products of alr1571 and 44% with ava_4178 respectively. The encoded proteins of alr1571 and ava_4178 share an amino acid sequence identity of 99%. Orthologues of alr0702 are found in many other cyanobacterial strains, but not always directly downstream of the *hyp*-operon. The transcript levels of the genes in the extended *hyp*-operon are low, since Northern blot analysis using specific probes within asr0689, alr0693, *hypF *or *hypAB*, failed to detect any mRNAs (data not shown).

### Transcriptional start point (TSP) analyses and characterization of the promoter regions

**Figure 2:**
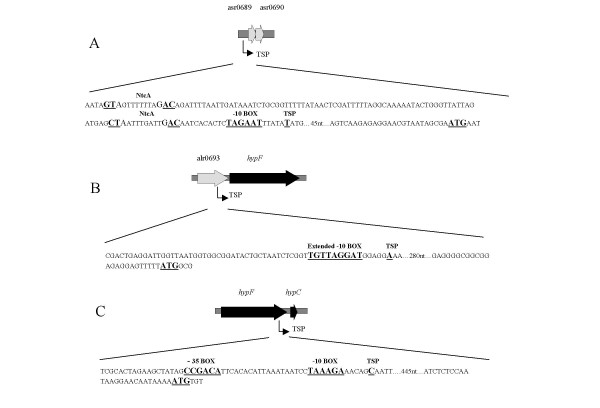
**Schematic presentation of the regulatory promoter regions of (A) asr0689, (B) *hypF *and (C) *hypC*.** Putative NtcA, -35, -10, extended -10, TSP and ATG sequences are enlarged and underlined.

5'RACE was performed to identify TSPs along the extended *hyp*-operon. Based on known TSPs in the *hyp*-gene cluster of *Nostoc punctiforme *PCC 73102 [22] the upstream regions of four genes, asr0689, alr0693, *hypF *and *hypC*, were examined. Three TSPs were identified (Fig. 2A–C). One TSP was identified 74 bp upstream asr0689, with a putative σ^70^-like -10 box sequence (TAGAAT) and two putative NtcA-binding sites, centred around -28 bp (CTAATTTGATTGAC) and -113 bp (GTAGTTTTTTAGAC) with respect to the TSP. The second TSP was found 307 bp upstream *hypF *with a putative, extended -10 box (TGTTAGGAT) [23]. A third TSP was identified 475 bp upstream *hypC *together with a putative σ^70^-like sequence, including both a -35 and a -10 box, (CCGACA N19 TAAAGA) upstream of the TSP, respectively. No TSP could be identified upstream alr0693.

### Repeats and Palindromic hairpins

In BLAST searches of the complete 14 kb extended *hyp*-operon, ten conserved short (11–23 nts) sequences (csR1–csR6) were identified in six intergenic regions (R1–R6) (Table 3, Fig. 1). Each of the conserved sequences is widely distributed (14 to 79 times) within the genome of *Nostoc *sp. strain PCC 7120. In addition, one of the conserved sequences (csR4), located in the intergenic region between *hypF *and *hypC*, is also present 26 times on the alpha plasmid (Table 3). csR5.1/csR5.2 appear twice in the intergenic region of asr0701-alr0702. This sequence has previously been identified in *Nostoc *sp. strain PCC 7120 as a LTRR (Long Tandemly Repeated Repetitive sequence) [24]. Two of the intergenic regions, *hupS*-asr0689 and alr0693-*hypF*, harbour three and two different conserved sequences, respectively. Simulations suggest that some of the conserved sequences; csR2, csR3.1, csR4 an csR5, might form palindromic hairpins, with ΔG melting energies predicted of -1.3 to -10.5 kcal mol^-1^. However, the conserved sequences csR1, csR3.2 and csR6, have no favourable energies for putative formations of secondary structures.

**Table 3:** Conserved sequences in the intergenic regions of the extended *hyp*-operon in *Nostoc *sp. strain PCC 7120 (Fig. 1A and Fig. 4))

Intergenic regions (R)	Conserved sequences (cs)	Length (nt)	Appearances in the genome	Hairpin formation energy ΔG (kcal mol^-1^)	Distance from translational start point (nt)
R1 *hupS*-asr0689	csR1.1: ttgtcagttgtcagttgtca	20	44	no hairpin	476
	csR1.2: ttgttagttgttag	14	11	no hairpin	455
	csR1.3: ccactgactactgac	15	14	no hairpin	391
R2 alr0691-0692	csR2: agacgcgatt(c/a)atcgcgtct	20	34	-10,5	49
R3 alr0693-*hypF*	csR3.1: ggagggtttccctcc	15	19	-6,1	81
	csR3.2: aacttttcaaga	12	21	no hairpin	60
R4 *hypF-hypC*	csR4: attgcgaattg	11	79 + 26 (alpha plasmid)	-1,3	49
R5 asr0701-alr0702	csR5.1: aaatccctatcagggattgaaac	23	32	-5,8	256
	csR5.2: aaatccctatcagggattgaaac	23	32	-5,8	186
R6 alr0702-all0703	csR6: taggggtgtaggggt	15	61	no hairpin	^a^

^a ^Intergenic region downstream the two ORFs alr0702 and all0703.

## Discussion

### Transcription of the extended hyp-operon

This study demonstrates that the *hyp*-genes in *Nostoc *sp. strain PCC 7120 may be trancribed as a single operon which is in accordance with results from other cyanobacteria [22,25]. Furthermore, the five genes localised directly upstream and the two genes downstream of the *hyp*-operon form transcripts with the *hyp*-genes (Fig. 1A–C). In addition to the TSP positioned upstream of asr0689, two TSPs were identified upstream of *hypF *and *hypC*, respectively (Fig. 2A–C), indicating that multiple short transcripts within the operon may exist. The position of the TSP upstream *hypF *is in agreement with results from *Lyngbya majuscula *CCAP 1446/4 [25]. In *Nostoc punctiforme *PCC 73102, no TSPs were detected in the upstream vicinity of either *hypF *or *hypC*. Instead, a TSP was identified upstream the NpR0363, an orthologue to alr0693, positioned upstream from *hypF *as in *Nostoc *sp. strain PCC 7120, with a putative NtcA-binding site and a -10 box [22]. No TSP was identified upstream alr0693 in *Nostoc *sp. strain PCC 7120. Transcripts of varying sizes derived from the same operon have been reported in cyanobacteria, e.g. within the *hox*-operons in *Nostoc *sp. strain PCC 7120, and in *Synechococcus *sp. PCC 7942 [26,27]. A putative σ^70^-like -10 box was identified upstream the first gene in the extended *hyp*-cluster, asr0689, together with two putative NtcA-binding sites centred around -28 bp and -113 bp upstream the TSP. The sequence (CTAATTTGATTGAC) does not perfectly conform with the consensus sequence (GT N10 AC) shown to be important for NtcA binding, but there are examples where NtcA has been shown to bind to imperfect NtcA consensus sites. The position of the putative NtcA binding site located closer to the TSP than the more common 41.5 bp could indicate that the binding site would be compatible with NtcA acting as a repressor [23,29,30]. The second putative NtcA binding site (GTAGTTTTTTAGAC) centred -113 bp upstream the TSP has a perfect match to the consensus sequence (GT N10 AC). In the case of NtcA depending promoters a recognizable -35 box is usually missing [22]. The activity of NtcA will most probably be dependent of various growth parameters, and the presence of other regulating proteins and or metabolites interacting with NtcA. The promoter region upstream *hypF *contains an extended -10 box (TGNTAN3T) which belongs to a subclass of *E. coli *promoters which functions without a -35 box [23]. In the promoter region upstream *hypC *both a putative -35 and a -10 box have been identified. The presence of TSPs upstream from both *hypF *and *hypC *can be coupled to the specific function of the respective proteins. HypF is involved in the synthesis of the CN-ligands, while HypC and the downstream Hyp proteins are active in the insertion of the metal atoms into the active site and in the stabilization of the protein complex [6,13]. The promoter regions of *hypF *and *hypC *are both localized within respective upstream genes. This may indicate that they are individually expressed as a result of the need for more detailed regulation of the amount of the proteins translated from their respective mRNA.

**Figure 3:**
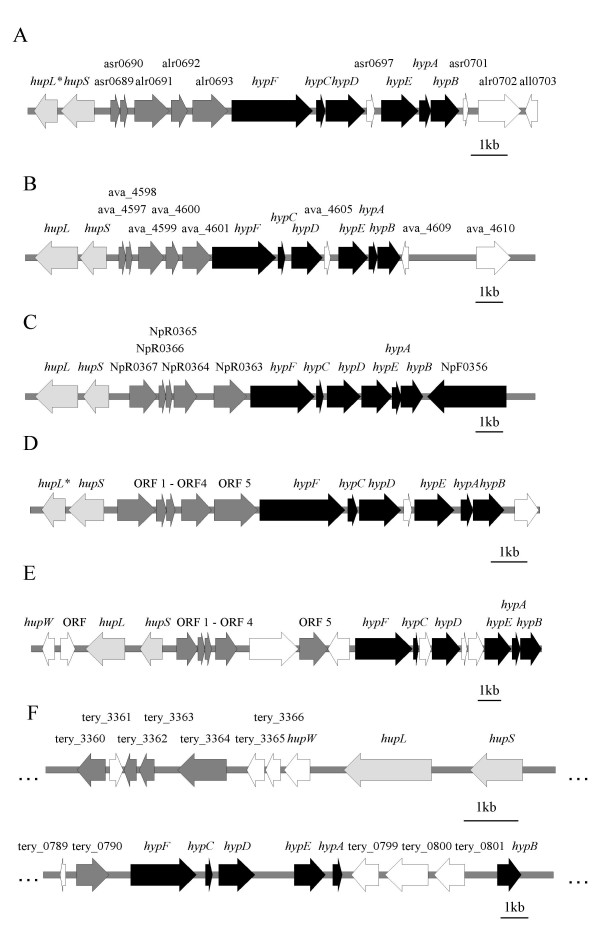
**Physical map of the genomic arrangement of the structural hydrogenase genes, *hupSL *(depicted in light grey), the putative maturation genes of the small subunit of the uptake hydrogenase (dark grey), and *hyp *genes (black) of filamentous nitrogen-fixing cyanobacteria.** (A) *Nostoc *sp. strain PCC 7120, (B) *Anabaena variabilis *ATCC 29413, (C) *Nostoc punctiforme *PCC 73102, (D) *Nodularia spumigena *CCY9414, (E) *Lyngbya majuscula *CCAP 1446/4, and (F) *Trichodesmium erythraeum *IMS101. The putative maturation genes of the small subunit of the uptake hydrogenase are located in close vicinity to the structural genes, *hupSL*, and often in between *hupSL *and the *hyp*-genes. In *Trichodesmium erythraeum *IMS101 *hupSL *and the putative maturation genes are separated from the *hyp*-gene cluster by approximately 3.8 Mb. *Indicates the N end fragment of the *hupL *(*hupL*5'), as annotated in vegetative cells.

The gene cluster asr0689-alr0693, positioned upstream of the *hyp*-operon (Fig. 1A–C) was shown to be transcribed together with the *hyp*-genes. These five ORFs are present in other filamentous, heterocystous N_2_-fixing cyanobacteria, e.g. *Anabaena variabilis *ATCC 29413 [31], *Nodularia spumigena *CCY9414 (AAVW01000003), and *Nostoc punctiforme *PCC 73102 [32]. Orthologues also exist in the filamentous, non-heterocystous N_2_-fixing cyanobacteria *Lyngbya majuscula *CCAP 1446/4 (AF368526) and *Trichodesmium erythraeum *IMS101 [34] (Fig. 3, Table 4). These ORFs are present in cyanobacterial strains containing an uptake hydrogenase but absent in strains harbouring only a bidirectional hydrogenase. In strains with these ORFs, the five identified ORFs are located between the *hupSL *and the *hyp*-genes, as in *Nostoc *sp. strain PCC 7120, with the exception of *Trichodesmium erythraeum *IMS101 where the *hyp*-genes are located ~3.8 Mb away from the structural *hup*-genes (Fig. 3).

**Table 4:** Orthologues to the five ORFs asr0689-alr0693 of *Nostoc *sp. strain PCC 7120 with conserved genomic arrangement in filamentous N_2_-fixing cyanobacteria (Fig. 3)

*Nostoc *sp. strain PCC 7120	asr0689	asr0690	alr0691	alr0692	alr0693	identical genomic arrangement
*Anabaena variabilis *ATCC 29413	ava_4597	ava_4598	ava_4599	ava_4600	ava_4601	identical genomic arrangement
*Nostoc punctiforme *PCC 73102	NpR0366	NpR0365	NpR0367	NpR0364	NpR0363	identical genomic arrangement
*Nodularia spumigena *CCY9414	ORF2	ORF3	ORF1	ORF4	ORF5	identical genomic arrangement
*Lyngbya majuscula *CCAP 1446/4	ORF2	ORF3	ORF1	ORF4	ORF5	identical genomic arrangement as the subgroup above, with the exception of additional ORFs within the cluster
*Trichodesmium erythraeum *IMS101	tery_3363	tery_3362	tery_3364	tery_3360	tery_0790^a^	identical genomic arrangement as the subgroup above, with the exception of additional ORFs within the cluster

^a ^Positioned directly upstream *hypF *with a distance of 3.8 Mb to *hupSL*.

There is not much known about the two ORFs, asr0689 and asr0690, except that they have putative membrane spanning regions and might function as ABC-transporters (Table 2). The protein encoded by alr0691 contains TPR domains (Tetratrico Peptide Repeat), which have been shown to be involved in functions as chaperone in protein-protein interactions and assembly of multi-protein complexes [34-36]. A relevant feature of the protein encoded by alr0692 is that it harbors a NifU-like domain partly overlapping a thioredoxin-like domain. NifU-like proteins show a high degree of similarity between species as different as humans and viruses, which suggests that they are much conserved [37]. Thioredoxins participate in redox reactions catalysing the reduction of intra-molecular disulfide bonds (IPR005746), and can play a role as sulphur donor in the mobilization of sulphur for maturation of (FeS) clusters [18]. The protein encoded by the fifth ORF alr0693 contains domains with TPR and NHL (NCL-1, HT2A and Lin-41) repeats. NHL repeats could, according to structural model analysis, be involved in protein-protein interactions (Table 2). The NHL domain is also associated with zinc finger motifs, which is often found in eukaryotes where they function as DNA binding motifs in transcription factors, by stabilizing a protein structure around the zinc atoms [38]. In *Nostoc punctiforme *sp. strain PCC 73102 NpR0363, the orthologue of alr0693, located in exactly the same position as in *Nostoc *sp. strain PCC 7120, is transcribed together with the *hyp*-genes with a defined TSP and a promoter region putatively controlled by NtcA [22]. A suggestion is that this ORF might be involved in the maturation process of the large subunit of the uptake hydrogenase together with the *hyp*-genes. The location is in accordance with the orthologue in *Trichodesmium erythraeum *IMS101, tery_0790, located directly upstream *hypF *and the *hyp*-genes, and has an identical arrangement as for the other orthologues of alr0693 (Fig. 3, Table 4).

### Maturation of the small subunit of the uptake hydrogenase

A study of the legume endosymbiont *Rhizobium leguminosarum *bv. Viciae st. UPM791 demonstrated that a cluster of four genes, *hupGHIJ*, positioned between the structural genes and the *hyp*-operon is involved in the maturation of the small subunit of the uptake hydrogenase [39]. Especially *hupG*, which has a structural domain related to thioredoxins and thiol-disulfide isomerases, and *hupH *which forms a complex structure with the pre-HupS seem to be important. HupH is thought to stabilize the protein complex as a chaperone during the maturation process and it has also domains characteristic of rubredoxins [39]. When using Blast-searches, no orthologues to the gene cluster *hupGHIJ *were found in the cyanobacterial genomes, but the two ORFs alr0691 and alr0692 contain conserved sequences encoding similar domains as present in HupH and HupG. Based on the finding that asr0689-alr0692 are transcribed together with the *hyp*-genes in *Nostoc *sp. strain PCC 7120 (Fig. 1), the existence of highly conserved orthologue regions in N_2_-fixing cyanobacteria positioned between *hupSL*, and the *hyp *genes, and that two of the ORFs alr0691 and alr0692, contain functional domains resembling *hupH *and *hupG *it is tempting to suggest that the upstream genes of the *hyp*-operon in *Nostoc *sp. strain PCC 7120 are involved in the assembly and maturation process of the cyanobacterial uptake hydrogenase small subunit. To prove if this hypothesis is true mutational studies followed by additional experiments will be done. Interestingly, orthologues to the gene cluster asr0689-alr0692 are absent in cyanobacteria harbouring only the bidirectional hydrogenase.

### Repeats and Palindromic hairpins

**Figure 4:**
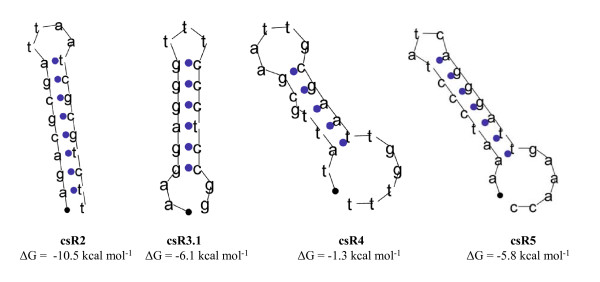
**The secondary structures formed by the repeated conserved sequences (cs) R2, R3.1, R4 and R5 found within the intergenic regions of the extended *hyp*-operon.** Predicted ΔG melting energies are shown for each putative hairpin structure. Conserved sequence R5 (csR5) is identical to the previously described LTRR in *Nostoc *sp. strain PCC 7120 [24].

In six of the intergenic regions of the extended *hyp*-operon, a total of ten kinds of conserved sequences were found, appearing between 11 and 79 times in the genome (Table 3). The conserved sequences that might form putative perfect palindromic structure (Fig. 4) could be involved in protein binding. The conserved sequence R5 (csR5) occurs twice in the same intergenic region (Fig. 4). Additionally, the intergenic region between *hypF *and *hypC *includes two csR4 sequences partly overlapping each other (**ATTGCGAATTG**CGAATTG). The conserved sequences are able to form putative hairpin secondary structures (Fig. 4) and are positioned between the transcriptional and the translational start point, which might indicate a function in translation. Another possibility is that these conserved sequences might have no functions at all and that they are results of evolutionary transposition events. To investigate the possible functions of the conserved sequences in the extended *hyp*-operon functional genomic experiments such as mutational studies will be performed.

## Conclusion

This study demonstrated that five ORFs encoding proteins with unknown functions are co-transcribed with the *hyp*-genes, and identified three TSPs, in *Nostoc *sp. strain PCC 7120 (Fig. 1). The additional conservation of these genes among N_2_-fixing cyanobacteria may indicate an important function, hypothetically in the maturation of the small subunit of the uptake hydrogenase.

## Methods

### Organisms and growth conditions

The filamentous heterocystous cyanobacterium *Nostoc *sp. strain PCC 7120 (also called *Anabaena *sp. strain PCC 7120) was cultured in BG11_0 _[40], sparged with air and grown at 25°C, with irradiance of 40 μmol of photons m^-2 ^s^-1 ^[41].

### Nucleic acid isolation and analysis

Genomic DNA was isolated from *Nostoc *sp. strain PCC 7120 cultures as described [42]. For total RNA isolation *Nostoc *sp. strain PCC 7120 cells were harvested by centrifugation, at 5,000 RPM, for 5 min, at 25°C, and incubated with TRIZOL reagent (Invitrogen), at 1 mL per 100 mg of cells. In the homogenization step, 0.5 g 0.6 mm diameter acid washed glass beads were added to the samples, and the disruption of the cells was accomplished using a PRECELLYS^®^24 lyser/homogeniser (Bertin Technologier). Phase separation was achieved by adding 0.2 mL chloroform per 1 mL added TRIZOL. RNA was precipitated by adding 0.25 mL 2-isopropanol and 0.25 mL high salt solution (0.8 M sodium citrate and 1.2 M sodium chloride). RNA was washed with 75% ethanol, adding at least 1 mL per mL TRIZOL reagent used initially. RNA was dried in air and resuspended in sterile water (RT-PCR experiments) or TE buffer (Tris-HCl EDTA pH 8.0, Northern blot hybridizations). The rRNA quality was analysed by agarose gel electrophoresis (1% agarose gel) using 0.5 × TBE buffer, and with the Experion System (Bio-Rad) according to the manufacturer's instructions. The concentration was determined by absorbance measurements at 260 nm (Cary Win UV, Varian).

### Primer construction

All oligonucleotides used are listed in Table 1. All primers, except the tag-primer, were designed by Primer3 program [43] and checked for their specificity by BLAST search against the *Nostoc *sp. strain PCC 7120 genome to check their specificity. The secondary structure was analysed with a Primer design utility program [44]. The tag-primer was designed using the program Tagenerator [21].

### Transcription analysis

Reverse transcription (RT) reactions were performed with the RevertAid™ H Minus First Strand cDNA Synthesis Kit (Fermentas), according to the instructions of the manufacturer using 2.5 μg total RNA from *Nostoc *sp. strain PCC 7120 cells grown under N_2_-fixing conditions. PCR amplifications using cDNAs of the respective genes were performed using corresponding primers (Table 1). Negative controls for the tag-primers were PCR on genomic DNA from *Nostoc *sp. strain PCC 7120 with the TAG and the respective forward primer. Positive controls were made with genomic DNA with the corresponding forward and reverse primer.

### PCR, DNA sequencing and sequence analysis

PCR amplifications were carried out using Eppendorf Mastermix (2.5×) (Eppendorf) in a MJ Mini™ Gradient Thermal Cycler PTC-1148 according to the guidelines provided by the suppliers. Obtained DNA fragments were isolated from the agarose gels using the GFX PCR DNA and Gel Band Purification Kit (GE Healthcare), following the manufacturer's instructions. Sequencing reactions were performed at Macrogen Inc. Computer-assisted sequence analyses were performed using BioEdit Sequence Alignment Editor Version 7.0.5.3.

### Identification of the transcription start point (TSP)

The TSPs were identified with the 5'RACE System for Rapid Amplification of cDNA Ends, Version 2.0 (Invitrogen) using 2.5 μg total RNA and gene specific primers (Table 1) following the manufacturer's instructions. Obtained PCR products were cloned into the pCR2.1-TOPO^® ^vector (Invitrogen) according to the manufacturer's instructions and sequenced. The experiment was repeated once.

### Northern blot hybridizations

Formaldehyde/denaturing gels were used to separate the total RNA, 7, 21 and 28 μg per lane, using the protocol provided together with the Hybond-N+nylon membrane (Amersham). Hybridizations were performed at 65°C, using probes obtained by PCR and genomic DNA from *Nostoc *sp. strain PCC 7120, for primer pairs used see Table 1. The experiment was repeated twice.

### In silico genome analyses of the extended hyp-operon

The search for conserved domains in the proteins with unknown functions were done in Cyanobase [45], Uniprot Knowledgebase (SwissProt and TrEMBL [46]), and Pfam [47]. The nucleotide sequence between *hupS *and all0703 checked for conserved sequences by BLAST search in Cyanobase [45]. In six of the intergenic regions conserved sequences were found, which were further analysed with CyanoBike [48] as well as studied manually. To identify putative secondary structures the sequences were analysed with mfold [49].

## Authors' contributions

ÅA performed most experimental work; 5'RACE, RT-PCR and in silico bioinformatics. She was the primary author of the final manuscript. KS supervised the experimental work and analyses of the data, and was also involved in all parts of the writing of the manuscript. MH carried out the Northern blots and was involved in the planning of the experiments and the analyses of the data. PL conceived and coordinated the project, and the manuscript. All authors have read and approved the manuscript.
